# Effects of Fermented Lingonberry Juice Mouthwash on Salivary Parameters—A One-Year Prospective Human Intervention Study

**DOI:** 10.3390/dj10040069

**Published:** 2022-04-14

**Authors:** Pirjo Pärnänen, Sari Lomu, Ismo T. Räisänen, Taina Tervahartiala, Timo Sorsa

**Affiliations:** 1 Department of Oral and Maxillofacial Diseases, Head and Neck Center, University of Helsinki and Helsinki University Hospital, P.O. Box 63 (Haartmaninkatu 8), FI-00014 Helsinki, Finland; sari.lomu@gmail.com (S.L.); ismo.raisanen@helsinki.fi (I.T.R.); taina.tervahartiala@helsinki.fi (T.T.); timo.sorsa@helsinki.fi (T.S.); 2 Division of Periodontology, Department of Dental Medicine, Karolinska Institute, 17177 Huddinge, Sweden

**Keywords:** fermented lingonberry juice, resting saliva, stimulated saliva, salivary buffering capacity, saliva pH, dry mouth, xerostomia

## Abstract

A one-year prospective human intervention study was performed to examine the effects of fermented lingonberry juice (FLJ), used as a mouthwash for six months, on salivary parameters. A total of 25 adult participants used 10 mL of FLJ as mouthwash 30 s daily for 6 months in addition to their normal oral homecare routines. Standard oral examinations and gathering of samples were performed at the beginning of the study and after six months and one year. Resting and stimulated saliva secretion rates, resting saliva pH, and stimulated saliva buffering capacity were determined. A questionnaire of participants’ subjective sensations of mouth dryness was also recorded at each timepoint. Fermented lingonberry juice mouthwash had positive effect to all five salivary parameters and were, according to the omnibus test, statistically significant during the study period. Analysis of the subjective dry mouth sensation questionnaires revealed that symptoms of xerostomia decreased due to the use of FLJ. This study revealed that the once-a-day use of FLJ mouthwash had a beneficial, increasing effect on salivary flow rates, buffering capacity, and salivary pH. FLJ thus can be safely used as an adjunctive and beneficial therapy in oral homecare, protecting teeth and oral mucosa, including periodontium, and also relieving dry mouth symptoms.

## 1. Introduction

Saliva has multiple functions in maintaining and controlling oral health. It protects and lubricates the oral mucosa, exerts buffering action against acidity variations after meals, takes part in the mineralization and demineralization of tooth enamel and dentin, has antimicrobial activity with its immunological and non-immunological components, as well enhances taste and begins the digestive process. Whole saliva is a mixture of water secretion produced by major salivary glands (parotid, submandibular, and sublingual), minor salivary glands (lower lip, tongue, palate, cheeks, and pharynx), and gingival crevicular fluid secretions [1] containing minerals, proteins and mucins. Diseases, such as diabetes mellitus, the autoimmune disease Sjögren’s syndrome, and medications or radiotherapy cause salivary gland dysfunction and may lead to xerostomia, altered dysbiotic microbial balance and dental caries, periodontal diseases, or mucosal lesions, such as candidosis [2,3,4,5,6]. Normal pH of saliva is 6–7, unstimulated saliva flow rate is 0.3–0.4 mL/min, the stimulated saliva flow rate 1–3 mL/min, and the buffering capacity 10–12. In hyposalivation the resting saliva flow rate is <0.1 mL/min, and the stimulated saliva flow rate < 0.7 mL/min. Eating or drinking causes alterations to saliva pH and secretion rates, and depending on beverage acidity, sugar content, and consumption frequency, buffering occurs in 5 to 15 min [7,8]. The non-microbial pellicle layer on teeth surfaces is an important intermediate in protecting and balancing environmental pH changes. The consumption of fermentable carbohydrates, acidic liquids, or sugar in the presence of acidogenic microbes causes the initiation of enamel demineralization under the critical pH 5.5 described by R.M. Stephan [9]. Saliva acts to counteract this process, but low buffering capacity and saliva pH predispose to erosion. There are limited means to regulate salivary flow. Topical management with lubricating gels, toothpastes, sprays, or artificial saliva may relieve sensations of dry mouth and xerostomia [10], acting as salivary stimulants or substitutes. Salivary-flow-inducing xylitol chewing gum or acidic substances eventually give temporary relief. Medications inducing salivary flow, such as systemically acting parasympatomimetic pilocarpine and cevimeline, exert side effects or are even contraindicated in patients with iritis, narrow-angle glaucoma, gastric ulcer, uncontrolled asthma, or in patients using β-blockers [11,12]. Several natural compounds have been evaluated for oral use. Lingonberries contain bioactive phenolic compounds with antioxidative, anti-inflammatory, and antimicrobial properties [13]. Based on previous in vitro and in vivo clinical human oral studies [14,15], FLJ used as mouthwash has revealed antimicrobial, anti-inflammatory, and anti-proteolytic effects, but the effects of lingonberries on salivary parameters have not been studied.

The aims of this prospective human intervention study were to evaluate the effects of FLJ on saliva secretion rates, saliva pH, and buffering capacity with basic clinical saliva test methods in randomly selected adult patients from two private dental clinics in Finland. The hypothesis of our study was whether fermented lingonberry juice, used as a mouthwash once a day in addition to oral homecare, could increase salivary flow and buffering capacity without decreasing pH, thus relieving symptoms of hyposalivation safely.

## 2. Materials and Methods

Twenty-five adults (28–91 years, mean 65.29 +/− 16.23 years; M/F ratio 10/15) were recruited randomly from two separate private dental clinics in Helsinki and Joensuu (Finland). The study took place from June 2020 to June 2021. Healthy patients or with controlled systemic diseases, good cognitive ability, users of removable dental prostheses (one patient with upper whole denture and three patients with upper partial denture), with health adequate periodontal tissue, with or without xerostomia, and without mucosal inflammation were included to the study. Medical evaluations revealed underlying diseases such as cardiovascular diseases and diabetes mellitus but no autoimmune diseases. Exclusion criteria were the use of other mouthwash products than FLJ, such as chlorhexidine, or current use of antibiotics during the study.

Resting saliva flow rate and pH, stimulated saliva flow rate, and buffering capacity were measured and analyzed by Saliva-Check BUFFER test kit (GC America Inc., Illinois, USA) at the onset and after 6 and 12 months. Patients were instructed not to eat, drink, or brush teeth for one hour prior to the examination appointment. Saliva collections were carried out in the afternoon. The patient was guided to swallow all saliva in the mouth, followed by resting saliva drain in a face down position into a cup without spitting or facial movements for 5 min, and asked to spit all saliva from the mouth at the end of the gathering. Resting saliva flow rate > 0.3 mL/min was considered normal, 0.1–0.3 low, and <0.1 mL/min extremely low. The Saliva-Check BUFFER test kit includes pH test papers and the buffer capacity measuring strips. The pH-paper and all four pads on the plastic buffer capacity strip were wetted thoroughly with resting saliva using a pipette and turned sideways on a paper towel to absorb the excess saliva. The color changes were compared to the specific color chart within suggested time limit provided by the manufacturer to obtain numerical values. Buffering capacity values (scale 0–12) of 0–5 were considered extremely low, 6–9 low, and 10–12 normal/high. Values of saliva pH (scale 5.0–7.8, scale interval 0.2) of 5.0–5.8 were considered highly acidic, 6.0–6.6 moderately acidic, and 6.8–7.8 normal [16]. Stimulated saliva secretion was induced by chewing wax and collected for 5 min. Stimulated saliva secretion rate >1 mL/min was considered normal, 0.7–1 mL/min low, and <0.7 mL/min extremely low. Diseases and medications were recorded.

Medications causing xerostomia (all classes from weak to strong effect pooled) were recorded according to Wolff et al. [2]. Additionally, a questionnaire for subjective sensations of xerostomia was filled at each timepoint (Table 1). Answers (yes/no) with indications of xerostomia were recorded, counted, and the points summarized for each patient.

Ten milliliters of FLJ (Lingora^®^, Vantaa, Finland) [14] was used by the participants as a mouthwash for thirty seconds daily for six months in addition to their daily oral homecare, following a six-month washout period without the FLJ mouthwash regimen. Phenolic compound concentration of the mouthwash was 0.219 % (*w*/*w*) and the pH was 2.77. The patients used nonprofessional toothpaste in oral homecare throughout the study, and professional topical fluoride was applied at each timepoint. This study has received approval from the ethical committee of Stockholm Community, Sweden (2016-08-24/2016/1:8 and 2016-1-24) and the Helsinki University Central Hospital, Finland (360/13/03/00/13 and 51/13/02/2009). Informed consent was obtained from all subjects involved in the study.

Salivary parameters (resting saliva, resting saliva pH, stimulated saliva, buffering capacity) and subjective dry mouth sensations from 21 subjects were measured at 3 time points (0, 6, and 12 months). The overall differences between the time points in the levels of each salivary parameter were determined with non-parametric Friedman’s test and pairwise post hoc analysis with Bonferroni corrected Wilcoxon signed-rank tests. SPSS (version 27; IBM Corp., Armonk, NY, USA); the rmcorr package (version 0.4.1) in the R statistical software version 3.6.3 was used in the statistical analyses, and *p* < 0.05 was considered as statistically significant. For one-way repeated measures analysis of variance, effect size = 0.40, α = 0.05, and 95% power for each group was considered to be appropriate for 18 participants. Previous studies regarding FLJ to calculate effect size were not available.

## 3. Results

**Figure 1:**
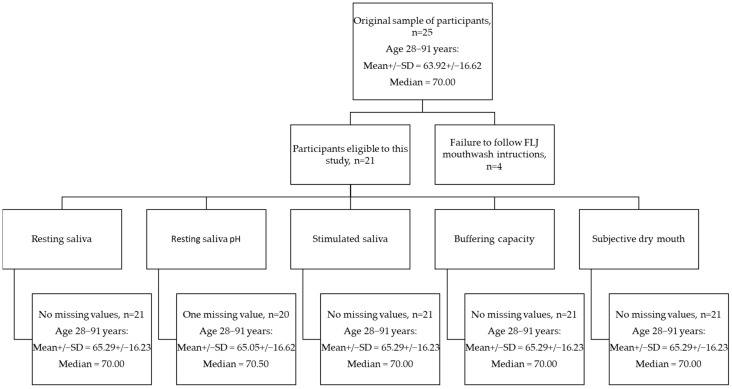
Flowchart of sample number and age of participants in the current study.

A total of 21 of the recruited participants used the mouthwash according to instructions (10 mL/once a day) and were included in the analyses. The number of the final sample and age of the participants may be seen in Figure 1. None of the participants discontinued the study. One patient had so low resting saliva secretion that the pH could not be measured. Four of the patients did not use FLJ as directed: One patient used FLJ mouthwash 5 mL/once a day, two used 10 mL irregularly, and one used 20 mL/once a day. In Table 2, patient characteristics, the specifications of the diseases, and the used medications with frequencies (%) among the participants are shown. The cohort selected for our study represents typical ambulatory middle-aged Scandinavian people visiting their dentists once yearly seemingly well.

Number of diseases belonging to endocrine and metabolic, mental and behavioral, circulatory system, and respiratory system were classified according to the International Statistical Classification of Diseases and Related Health Problems [17]. The number of medications reported with higher or moderate level of evidence to induce xerostomia included medications for endocrine and metabolic (diabetes), the circulatory system (high blood pressure), and mental and behavioral diseases [2].

**Figure 2:**
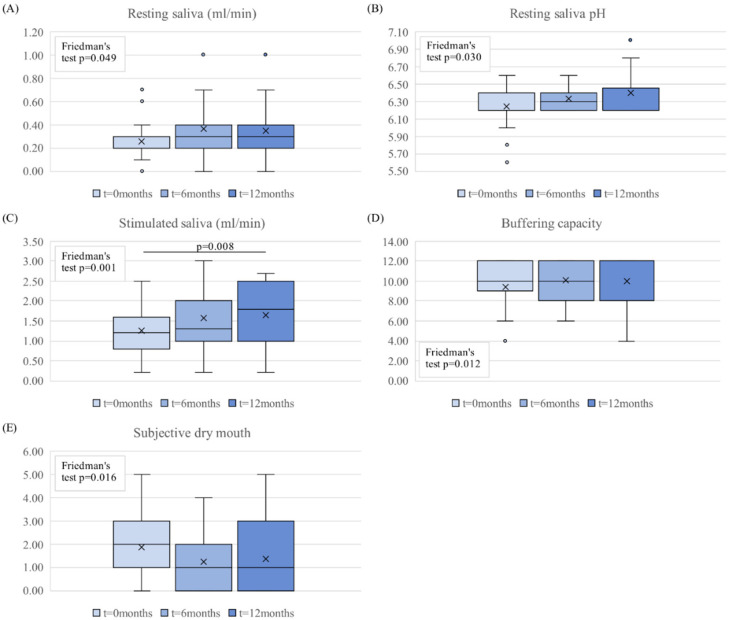
The boxplots of the salivary parameters (**A**) resting saliva, (**B**) resting saliva pH, (**C**) stimulated saliva, (**D**) buffering capacity, and (**E**) subjective dry mouth sensations for 3 time points of 0, 6, and 12 months (n = 21). The omnibus analysis of these parameters at three timepoints was conducted with Friedman’s test and pairwise post hoc analysis with Bonferroni corrected Wilcoxon signed-rank tests. The boxplots show the median, mean (x), quartiles, and extreme values (o).

The results of the salivary parameters are shown in Figure 2. The FLJ mouthwash had positive effect on all five salivary parameters, and according to the omnibus test, all parameters were statistically significant during the study period (three measuring points).

- Resting saliva flow rate increased from low to normal levels during the FLJ period and remained at this level during the washout period (Figure 2A);
- Resting saliva pH increased progressively during the trial (Figure 2B);
- Stimulated saliva flow rate increased during the FLJ period and continued to increase during the washout period (Figure 2C);
- Buffering capacity increased from near-low values to normal values during the FLJ period and stayed at normal level during the washout period (Figure 2D);
- In the beginning of the study, there was a small correlation between patient’s subjective dry mouth symptoms and resting and stimulated salivary flow, resting saliva pH, and buffering capacity (r = −0.432, *p* = 0.05; r = −0.482, *p* = 0.027; r = −0.357, *p* = 0.123; and r = −0.287, *p* = 0.207; respectively). The results from the questionnaire showed that subjective dry mouth symptoms decreased during the FLJ mouthwash period, kept to lower levels during the washout period compared to the beginning of the study, and had a negative correlation with resting and stimulated saliva flow rates and resting saliva pH. In other words, the increase in these parameters decreased the sensation of mouth dryness;
- Frequencies (N) of participants in each classification of variables during the study are shown in Table 3.

Noteworthy, 4 respondents out of 25 did not obey the instructions provided, and their buffering capacities had no statistically significant changes. No erosive lesions or mucosal irritation were noticed during the intervention, and no adverse effects were reported from FLJ mouthwash by the participants.

## 4. Discussion

Sufficient salivary flow rates, buffering capacity, and pH are crucial factors in protecting the oral mucosa and the teeth. The sugar clearance time, buffering capacity, and pH of saliva decrease with flow rate [18]. Reduced salivary pH, flow rate, and buffering capacity have been found from patients suffering from diabetes mellitus or asthma and chronic obstructive pulmonary disease (COPD) [19,20]. Alteration in salivary parameters also from older adults, adolescents, and xerostomia have been associated with higher prevalence of caries, periodontal disease, and candidosis [5,21]. The autoimmune disease, Sjögren’s syndrome, causes oral symptoms related to decreased saliva secretion [22]. To our best understanding, our cohort represented a characteristic and representative ambulatory set of the Scandinavian middle-aged population in respect to age, habits, weights, sex, diseases, and their medications. Sensations of dry mouth were correlated with resting and stimulated saliva flow rates and resting saliva pH in the current study. Stimulating saliva secretion is one of the main treatment options in relieving these symptoms [23]. The results from the current study reveal significant increases in the stimulated saliva flow rate and resting saliva pH and flow rate and salivary buffering capacity clearly increased during the FLJ mouthwash period.

The current study shows that FLJ has a sustained beneficial effect in maintaining safe saliva pH, and this is a beneficial effect compared to CHX, which in fact has been shown in another study to decrease saliva pH and buffering capacity [24], which might lead to more acidic conditions for potential periodontopathogens and oral microbes to thrive. The remineralization of enamel and dentin occurs if the exposure time is short enough to overcome demineralization [25]. Liquids are buffered faster compared to solids. The role of MMPs in dental caries and erosion has also been proposed, and inactivation of MMP-8 with chlorhexidine, FeSO_4_ and green tea has been shown to inhibit dental erosion development [26,27]. Fermented lingonberry juice has anti-inflammatory [13,15] and remarkably similar antimicrobial and anti-proteolytic effects as CHX exerts [14,15,28,29], and indeed, our previous in vitro study extended and further confirmed these findings, revealing that FLJ can inhibit *Candida glabrata* cell-wall-induced activation of pro-MMP-8 [30].

Several in vitro studies have shown that lingonberry phenolic compounds exert antibacterial [31,32,33], antiviral [34], antioxidative [35,36,37,38,39,40,41,42], anti-inflammatory [30,35], and anticancerous effects [43,44,45,46]. In vivo mouse studies show that lingonberry juice has anti-atherothrombic and anti-inflammatory effects, and lingonberries reduce hyperglycaemia, high cholesterol, obesity, improve metabolic/brain functions, and reduce gut inflammation [47,48,49,50]. Lingonberries, lingonberry puree, and lingonberry nectar have shown in vivo effects on glucose, insulin, and free fatty acid release responses in humans [51,52]. Oral human in vivo effects with FLJ show reduced *S. mutans* and *Candida* counts, as well as anti-inflammatory effects [15]. Saliva composition and volume have an important role in lubricating the mucosa and protecting from infections as part of the innate immunity, and it has been proposed that the glycoprotein profile of mucins may have a role in the regulation of the infection spreading and could even be used as a biomarker to predict disease susceptibility, disease progression, and response to therapy of COVID-19 patients [53]. Microbial proteases may modify these glycoproteins and cause pathogen entry. Lingonberry polyphenols show several antiviral mechanisms in vitro [54].

Sufficient saliva secretion has a pivotal role in the oral environment, and keeping these beneficial effects of lingonberries in mind, FLJ is designed for safe oral use in addition to oral homecare and may be used safely for long periods of time. Because FLJ juice is all-natural and considered similar to lingonberry juice, general recommendations regarding daily consumption of berries and berry juices are considered safe: 10 mL of FLJ is equal to appr. 1 dl of lingonberries; the consumption of berries, fruits, and vegetables are recommended 250 g/day in a healthy diet [55]. It has no additives, may be swallowed, and has no known interactions with medications and no side-effects. Due to the fermentation process, it contains only a fraction of naturally occurring sugars as compared to unfermented lingonberry juice, and the recommended amount once daily has positive effects on the saliva parameters. In the current study, topical fluoride application at each sampling time point was controlled professionally, but no specific toothpaste product was provided to the participants by the researcher, and this might be a limitation of the study.

Attempts to develop products to alleviate dry mouth symptoms are based on several mechanisms, e.g., protection of the mucosa by replacing missing saliva by artificial saliva, moisture-binding surface agents, mechanically increasing saliva secretion by chewing gums or drug-related routes. They often have only short-term effects or side-effects and are not actually sialagogues. Natural products have gained interest in the treatment of xerostomia [56]. The downside with most of these products is that they are based on acids, which pose a risk for mucosal irritation and have adverse effects on dentition due to the decrease in saliva pH or are incapability of inducing salivary flow [57]. The precise mechanisms of the effects of FLJ on salivary parameters are not precisely known. Potential mechanisms may include the induction of salivary gland saliva secretion by acids and resulting increase also in saliva pH and buffer capacity. The complex FLJ polyphenol composition has additional mucous membrane protective antioxidant, anti-inflammatory, anti-proteolytic, and antimicrobial effects, which may contribute to saliva properties via the effects on oral microbiota composition and its metabolic products.

## 5. Conclusions

The results of this prospective human intervention study demonstrated for the first time, that a daily regimen of 10 mL of FLJ for 6 months improved saliva secretion rates, saliva buffering capacity, increased saliva pH, and decreased xerostomia These effects continued and were monitored even at the end of the six-month washout period. These positive effects of FLJ on saliva parameters also make it a potential natural aid in relieving dry mouth symptoms safely. Considering the importance of salivary parameters also on the risk for caries, periodontal diseases and candidosis, larger randomized placebo-controlled studies are warranted to confirm our results.

## Figures and Tables

**Table 1:** Xerostomia sensations questionnaire.

1. Does your mouth feel dry after eating? 2. Do you have difficulties in swallowing? 3. Are you able to eat dry bread or biscuit without drinking? 4. Do you feel your saliva secretion is low? 5. How often do you wake up at night because of dry mouth sensations?

**Table 2:** Patient characteristics.

Age (mean ± standard deviation) Gender (female/male,%) Smoking (yes%) Diseases (mean, range) Medications (mean, range) Medications inducing xerostomia (mean, range)	65.29 ± 16.23 years 61.9/38.1% 19.0% 1.76, 0–4 2.95, 0–9 1.33, 0–4

**Table 3:** Frequencies of participants in each classification of variables during the study.

**Saliva sampling (months)**	**0**	**6**	**12**
**Resting saliva flow (N)**			
Extremely low (<0.1 ml/min)	4	2	3
Low (0.1–0.7ml/min)	13	15	14
Normal (>0.3 ml/min)	8	8	8
**Stimulated saliva (N)**			
Extremely low (<0.7 ml/min)	2	1	2
Low (0.7–1 ml/min)	10	8	6
Normal (>1 ml/min)	13	16	17
**Resting saliva pH (N)**			
Highly acidic (5–5,8)	2	0	0
Moderately acidic (6–6.6)	20	24	20
Healthy (6.8–7.8)	2	0	4
**Buffering capacity (N)**			
Very low (0–5)	2	0	1
Low (6–9)	7	8	5
Normal/high (10–12)	16	17	19

## Data Availability

Data supporting reported results can be obtained from the authors on request.
